# Patient Voice and Treatment Nonadherence in Cancer Care: A Scoping Review of Sentiment Analysis

**DOI:** 10.3390/nursrep16010018

**Published:** 2026-01-08

**Authors:** Leon Wreyford, Raj Gururajan, Xujuan Zhou, Niall Higgins

**Affiliations:** 1 School of Business, University of Southern Queensland, Ipswich, QLD 4300, Australia; raj.gururajan@unisq.edu.au (R.G.); xujuan.zhou@unisq.edu.au (X.Z.); niall.higgins@health.qld.gov.au (N.H.); 2 The Park—Mental Health and Specialised Services, West Moreton Health, Brisbane, QLD 4076, Australia

**Keywords:** discourse analysis, medication concordance, natural language processing, nursing informatics, oncology, patient–clinician communication, person-centred care, sentiment analysis, treatment adherence

## Abstract

**Background:** Treatment nonadherence in oncology is common. Surveys often miss why patients do not follow recommendations. We synthesised Natural Language Processing (NLP) studies, mainly sentiment analysis, of patient-generated content (social media, forums, blogs, review sites, and survey free text) to identify communication and relationship factors linked to nonadherence and concordance. **Methods:** We conducted a scoping review (PRISMA-ScR). Searches of PubMed, CINAHL, and Scopus from 2013 to 15 June 2024 identified eligible studies. We included 25 studies. Data were charted by source, cancer type, NLP technique, and adherence/concordance indicators, then synthesised via discourse analysis and narrative synthesis. **Results:** Four themes emerged: (1) unmet emotional needs; (2) suboptimal information and communication; (3) unclear concordance within person-centred care; and (4) misinformation dynamics and perceived clinician bias. Sentiment analysis helped identify emotions and information gaps that surveys often miss. **Conclusions:** Patient-voice data suggest practical actions for nursing, including routine distress screening, teach-back, misinformation countermeasures, and explicit concordance checks to improve adherence and shared decision making. **Registration:** Not registered.

## 1. Introduction

Treatment nonadherence in oncology remains a persistent challenge, especially with the growing use of oral anticancer medications (OAMs) administered outside the clinic, which shifts daily responsibility to patients and elevates adherence risk [1]. Although adherence is widely recognized as a multi-factorial construct involving patient and health system dimensions [2,3], the field has relied heavily on structured questionnaires and numeric scores that often fail to illuminate why patients deviate from recommended regimens.

Conventional patient-reported measures present several limitations. Many studies do not distinguish intentional from unintentional nonadherence; this has been described as a distinction that matters for the design of targeted interventions [4]. Survey instruments can be affected by recall bias and acquiescence, and closed-question formats compress experience into fixed categories, obscuring the nuanced reasons behind patient dissatisfaction or discordance with care [5,6,7]. In addition, small or selective samples reduce generalizability, while conceptualizations of “satisfaction” vary, and timelines for acting on feedback can be protracted [8,9]. These constraints limit visibility into the emotional, informational, and relational factors that shape concordance and adherence [10,11,12].

Natural Language Processing (NLP), particularly sentiment analysis of patient-generated narratives, offers a complementary lens on the lived experience of cancer care. Analyses of social media, online support groups, blogs, public review sites, and free-text responses can bring to the surface what have otherwise been “invisible” patient-reported concerns at scale, providing a crowd-validated window into what matters most to patients [13,14]. Compared with structured surveys, sentiment and related text analytics interrogate the tone, polarity, and emotion of language, capturing subtle affective states and informational gaps [15,16]. Prior work has leveraged these approaches to characterize longitudinal emotion dynamics and the role of emotions in adherence behaviours. Examples include hormonal therapy for breast cancer [17] and extraction of patient perspectives across platforms and conditions [18,19,20]. Recurrent emotional constructs (e.g., fear, sadness, disgust, surprise) and perceptions of clinician bias or conflicting information have been identified as salient correlates of dissatisfaction, discordance, or withdrawal [21,22,23]. At the same time, patient empowerment in online communities underscores substantial informational needs that may exceed generic emotional support, pointing toward specific communication targets for teams and services [24].

Placing these insights within patient/person-centred care (PCC) highlights the importance of shared decision-making, goal alignment, and relationship quality [25,26,27,28]. If emotions, information needs, and perceptions of communication are key determinants of whether recommendations are accepted, acted upon, and sustained, then synthesizing the “patient voice” from unstructured text should help reveal actionable levers for nursing practice, education, and service design.

### Aim and Research Questions

This scoping review synthesizes evidence from NLP studies of patient-generated narratives (with an emphasis on sentiment analysis) to illuminate how emotions, information needs, and perceptions of communication relate to treatment nonadherence and concordance. Guided by discourse analysis, we address the following:

1. How can secondary data from NLP studies of patient narratives reveal factors influencing cancer-patient nonadherence that are missed by traditional surveys [13,14]?
2. Which patient-side themes (emotional, informational, relational) consistently emerge that can inform nursing strategies to improve communication, concordance, and adherence [21,23,24]?

## 2. Method

### 2.1. Design and Reporting

We conducted a scoping review to map and synthesise evidence on patient-generated oncology narratives analysed using Natural Language Processing (NLP), with emphasis on sentiment analysis. Reporting follows the PRISMA-ScR guideline [29]. Given the anticipated heterogeneity of data sources, analytic pipelines, and outcomes, we complemented PRISMA-ScR with the SWiM guidance for transparent narrative synthesis (nine items). The review questions and eligibility criteria were defined a priori and aligned to the review aim (communication, relationship, and information related determinants of adherence/concordance detectable in the patient voice). A scoping review was chosen to map a heterogeneous evidence base spanning platforms, analytic families, and outcomes, where meta-analysis was neither appropriate nor feasible.

### 2.2. Operational Definitions

Adherence: Extent to which patient behaviours (e.g., dosing, attendance) match agreed recommendations.

Concordance: Agreement achieved via SDM, aligning care with patient goals and values. Concordance differs from compliance: compliance implies unilateral instruction-following and is considered outdated in nursing, whereas concordance emphasises partnership and shared decisions.

### 2.3. Eligibility Criteria

#### 2.3.1. Inclusion

Studies were eligible if they (i) analysed patient-generated text related to cancer using NLP or sentiment analysis (e.g., social media posts, online support communities, blogs, public review platforms, survey free text responses); (ii) reported findings relevant to treatment adherence, nonadherence, or treatment concordance/discordance (definitions below); (iii) were peer reviewed journal articles or full peer reviewed conference papers; and (iv) were published in English between 2013 and 15 June 2024. The period from 2013 onwards was chosen because it marks the emergence and rapid development of Natural Language Processing (NLP) and sentiment analysis techniques in health research. Prior to 2013, such applications in oncology and patient-generated data were rare. From 2013, there was a notable increase in published studies using advanced NLP methods to analyse patient narratives, especially on social media and digital platforms. Ending in early 2024 ensures the review captures the most recent advances and publications, providing a comprehensive and up-to-date synthesis of the literature.

#### 2.3.2. Operational Definitions

Patient-generated text refers to content authored by adult patients themselves (i.e., individuals self-identifying as living with cancer) in public or semi-public online environments or in survey free text fields. Content authored by caregivers, family members, clinicians, journalists, or institutions was excluded unless the text was explicitly written from the patient’s first person perspective and attributable as such. Relevance to adherence/concordance was established through a two-step procedure: (a) keyword screening for concepts such as adherence, nonadherence, concordance, compliance, persistence, discontinuation, withdrawal, refusal, skipping doses, dose reduction, stopping treatment, shared decision making (SDM), goals/values alignment, and (b) contextual/thematic confirmation that the text or study findings linked these concepts to treatment-taking behaviour, decision making, or participation in recommended care.

#### 2.3.3. Exclusion

We excluded studies that (i) had no cancer focus; (ii) analysed content produced primarily by carers/children/adolescents rather than adult patients (unless written from the patient’s perspective as defined above); (iii) were economic evaluations, editorials, protocols, or non-empirical commentaries; (iv) were non English; or (v) constituted grey literature, with the exception of policy/ethics statements (e.g., WHO, ACSQHC) used for definitional clarity [2,30].

### 2.4. Information Sources and Search Strategy

We searched PubMed/MEDLINE, CINAHL, and Scopus for records from 2013 to mid-2024. The last search was performed on (15 June 2024). To maximize sensitivity for studies where titles/abstracts may not co-list all constructs, we built concept blocks around (1) cancer/neoplasms, (2) NLP/sentiment/text mining, (3) patient generated narratives/platforms, and (4) adherence/concordance/SDM constructs, and combined them with Boolean operators. Database specific syntax and field tags were adapted accordingly. Full database strings, including example blocks such as:

- Concept A (cancer): (cancer* OR neoplasm* OR oncolog*)
- Concept B (NLP/SA): (“natural language processing” OR “text mining” OR sentiment OR emotion* OR classifier* OR lexicon* OR transformer*)
- Concept C (patient voice/platforms): (“social media” OR Twitter OR Reddit OR forum* OR blog* OR “patient review” OR “free text”)
- Concept D (adherence/concordance): (adheren* OR nonadheren* OR concordan* OR complian* OR persistence OR discontinu* OR “shared decision*”)

To identify additional applications or cross disciplinary work, we ran supplementary searches in Google Scholar and screened reference lists of included records (backward citation chasing) and cited articles where relevant (forward citation chasing). Search results were exported to a reference manager/spreadsheet, and duplicates were removed using automated matching and manual verification.

#### Automation Sensitivity

Because some platforms are susceptible to automated/bot content and because bot-detection performance varies by method and language, we recorded where source platforms might be affected and treated such findings cautiously, consistent with prior corpus work in oncology narratives [13,15]. When included studies reported bot-filtering or authenticity checks, we noted their approach in the characteristics table.

### 2.5. Study Selection

**Figure 1:**
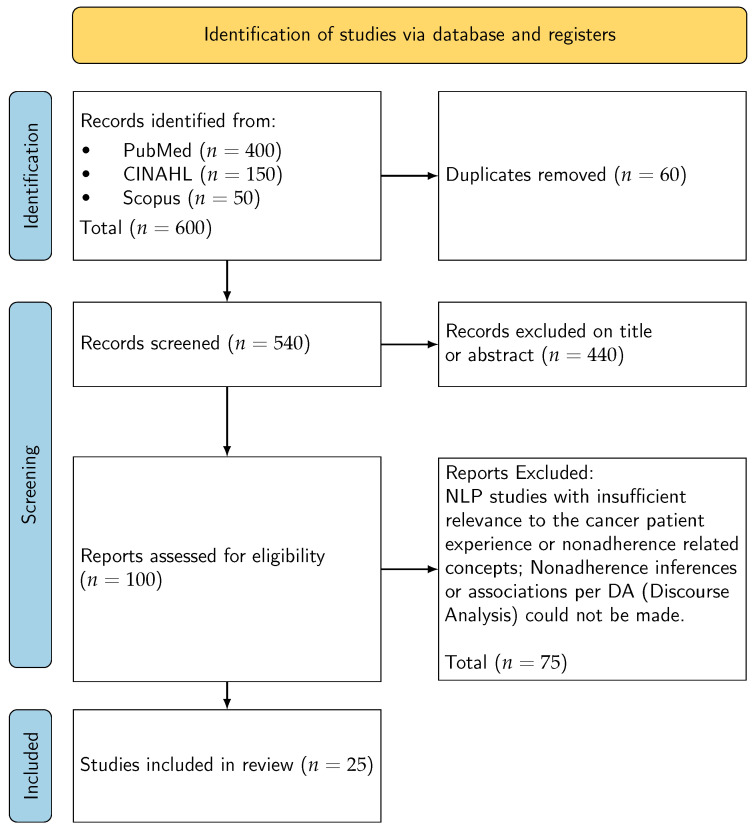
PRISMA flow diagram [29].

Two reviewers independently screened titles and abstracts against the eligibility criteria. We conducted a calibration exercise on an initial sample to standardise the application of inclusion/exclusion rules. Potentially eligible records underwent full text assessment by the same reviewers. Disagreements were resolved through discussion; a third reviewer was available for arbitration if consensus could not be reached. We conducted a pilot calibration and did not compute a formal kappa; consistency was assured via consensus with escalation to the third reviewer when required. Reasons for exclusion at the full text stage were documented. The study selection process is depicted in the PRISMA-ScR flow diagram (Figure 1) [29].

### 2.6. Data Charting

We developed and piloted a standardized charting form aligned to the review questions. Extracted items included the following: author/year/country; cancer type/domain; data source and platform (e.g., Twitter, Reddit, forums, blogs, survey free text); sampling frame/size; NLP/sentiment methods (e.g., lexicon/rule based, supervised models, transformer architectures; emotion vs. polarity outputs); key analytic parameters (e.g., lexicons used, model transparency); reported adherence/concordance indicators and how they were operationalized; and main findings relevant to emotions, information, and relationships. The form was iteratively refined after piloting. Charted data informed a characteristics table and thematic synthesis of patient side constructs.

### 2.7. Critical Appraisal

Given the heterogeneity of study designs (observational NLP analyses; mixed methods; qualitative components), we used the Critical Appraisal Toolkit (CAT) to describe methodological rigour across sources [31]. Appraisal focused on clarity of research question, appropriateness and transparency of data sources and NLP pipelines, sampling limitations and platform biases, validity of adherence/concordance inferences, and ethical handling of online content. Ratings (e.g., low/medium/high) and brief justifications were recorded to inform interpretation rather than to exclude studies, consistent with scoping review practice [32,33].

### 2.8. Ethical Considerations for Online Patient-Generated Data

We followed the Association of Internet Researchers’ ethics principles (contextual integrity, harm minimization) and relevant clinical communications guidance [30,33]. We restricted attention to public or clearly semi-public spaces; private groups or closed forums requiring membership approval were not targeted as primary sources. Where studies quoted user content, we favored paraphrased reporting in this review to reduce traceability via search engines. No attempts were made to identify or re-identify individuals. Platform terms of service and community guidelines cited by included studies were noted where available. The review used only published, aggregated findings from included sources and did not involve intervention or contact with users; institutional ethical review was therefore not required for this synthesis.

### 2.9. Synthesis Approach

**Figure 2:**
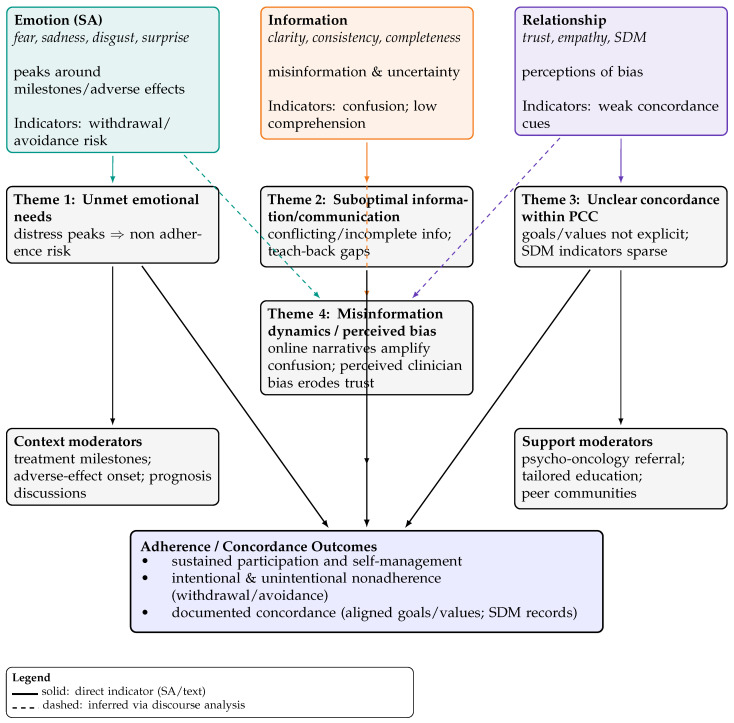
Conceptual links between sentiment-derived constructs (emotion, information, relationship) and adherence/concordance phenomena, grouped into patient-side themes by narrative synthesis ([15,17,23,24]; discourse-analysis inference: [21,22]). Abbreviations: SA—sentiment analysis; SDM—shared decision making; PCC—person-centred care.

We employed a two-stage synthesis. First, we mapped included studies by platform, cancer domain, and NLP approach to describe the evidence landscape. Second, we undertook narrative synthesis, integrating discourse analysis to link sentiment-derived constructs (emotion, information clarity, relationship/trust/SDM) to adherence and concordance phenomena [21,22]. To enhance transparency (SWiM), we pre-specified groupings by platform (Twitter/X, Reddit, forums/blogs, survey free text), analytic family (lexicon/rule based vs. supervised/transformer), and cancer domain; we summarized outcomes using standardized polarity/emotion metrics where available and otherwise used structured textual summaries. We explored heterogeneity by platform and method and prioritized results by analytic transparency and sample size. Visual synthesis is provided via a conceptual map (Figure 2), indicating whether links were directly observed in sentiment outputs or inferred through discourse analysis.

### 2.10. Synthesis Without Meta Analysis (SWiM)

In line with SWiM, we (i) specified the synthesis objective (identify communication, information, and relationship related determinants of adherence/concordance emergent from patient narratives); (ii) defined grouping variables (platform, cancer domain, NLP family); (iii) stated outcome handling (polarity/emotion classes, concordance/adherence indicators); (iv) described the synthesis method (structured narrative plus discourse analysis); (v) addressed limitations of combining diverse metrics (no quantitative pooling; emphasis on direction and consistency of effects/signals); (vi) examined heterogeneity qualitatively (by platform/method and appraisal ratings); (vii) considered study appraisal in interpretation (down weighting weakly reported inferences); (viii) used visual displays (PRISMA diagram; conceptual map). Reporting complements PRISMA-ScR to support reproducible narrative synthesis [29].

## 3. Results

### 3.1. Study Selection and Overview

From an initial corpus spanning PubMed, CINAHL, and Scopus (2013–early 2024), 25 studies met the inclusion criteria for analysis of patient-generated cancer narratives using NLP or sentiment analysis. Reasons for exclusion included non-cancer focus, non-English language, content authored by carers or children, and grey literature outside defined exceptions. The study selection process is illustrated in the PRISMA flow diagram (Figure 1).

### 3.2. Characteristics of Included Studies

The included studies varied in cancer type, data source (e.g., Twitter, forums, blogs, survey free text), and NLP technique (e.g., rule-based filtering, transformer models, emotion classification). Table 1 summarizes these characteristics, highlighting the adherence or concordance indicators most relevant to nursing practice.

### 3.3. Reliability and Validation of NLP Methods

The included studies employed a range of NLP techniques, such as lexicon-based sentiment analysis, machine learning classifiers, and transformer models like BERT. Methodological rigour varied: some studies reported validation of their algorithms against human-coded data, while others relied solely on automated outputs. For example, several sentiment analysis studies used manual annotation or expert review to confirm that the AI correctly identified emotions such as “fear” or “sadness” in patient narratives (e.g., [21,22]). However, not all studies provided detailed information on validation procedures or inter-rater reliability.

For a nursing audience, it is important to note that the reliability of NLP findings depends on whether human validation was performed. Studies that included manual checks or expert review of algorithmic outputs offer greater confidence that the AI accurately captured clinically relevant signals. In contrast, results from unvalidated algorithms should be interpreted with caution, as misclassification of emotions or adherence behaviours is possible.

Overall, while NLP methods show promise for extracting meaningful insights from patient narratives, future research should prioritise transparent reporting of validation procedures and methodological rigour to ensure findings are robust and clinically applicable.

### 3.4. Emergent Patient-Side Themes

We distinguish two evidence tiers: (a) measured adherence, where sentiment or text signals were linked to observed adherence metrics (e.g., claims or documented completion [43]); and (b) inferred adherence, where emotions or communication issues were associated conceptually with nonadherence risks. The narrative below treats measured links as stronger evidence and labels inferred links accordingly. Unmet emotional needs (inferred; measured in subset): Emotions such as fear, sadness, disgust, and surprise often peaked around treatment milestones and adverse effects, aligning with withdrawal or intentional nonadherence in measured subsets while suggesting risk in other contexts. Suboptimal information and communication (inferred): Conflicting or incomplete information, including misleading framings (e.g., “good cancer”,) contributed to confusion and reduced trust in care teams. Unclear concordance within person-centred care (inferred): Patient narratives frequently lacked explicit SDM indicators or goal alignment, indicating missed opportunities for concordance checks. Misinformation dynamics and perceived clinician bias (inferred): Online narratives amplified confusion and sometimes reflected perceived bias, eroding trust and complicating adherence. These themes are visualized in the conceptual map (Figure 2), which links sentiment-derived constructs (emotion, information, relationship) to adherence outcomes via direct and inferred indicators.

### 3.5. Theme Prevalence Across Studies

Table 2 reports the prevalence of each theme across the included studies. Suboptimal communication and unclear concordance were the most frequently observed, followed by emotional distress and misinformation dynamics.

### 3.6. Narrative Summary

Across platforms, emotional distress was a consistent signal of nonadherence risk. Informational gaps and conflicting messages undermined understanding and trust, while sparse evidence of SDM pointed to missed opportunities for concordance. Online misinformation and perceived bias further complicated the communication landscape. These findings suggest that integrating emotion detection, teach-back strategies, and concordance checks into nursing workflows may improve adherence and patient engagement.

## 4. Discussion

To improve readability, we consolidate key insights across subsections below, focusing on evidential strength (measured vs. inferred), platform differences (social vs. EHR), and practical implications for nursing. This scoping review synthesizes findings from 25 studies that used primarily NLP-based sentiment analysis to explore cancer patient treatment nonadherence and discordance. The review identifies four key themes: unmet emotional needs, suboptimal communication and information, unclear concordance in Patient-Centred Care (PCC), and EHR barriers and compliance design.

### 4.1. Linking Findings to Review Questions

The review addressed two questions: (1) how secondary data from NLP studies can reveal insights hidden from traditional surveys, and (2) how oncologist narratives in EHRs reflect communication factors linked to nonadherence. The findings suggest that NLP techniques, particularly sentiment analysis, can uncover emotional and relational dimensions of the patient experience that are often overlooked in conventional research [13,14].

### 4.2. Conceptual Contributions

This review contributes to the understanding of treatment nonadherence by showing that discordance is more frequently inferred than directly measured. Emotional constructs such as fear, sadness, and spiritual pain were detected in patient-authored content but rarely documented in EHRs [22,35]. The review also highlights the potential of NLP to detect fine-grained emotional states and communication breakdowns that influence adherence.

### 4.3. Platform and Methodological Insights

Sentiment analysis of social media and online support groups revealed richer emotional content than text mining of EHRs, which often lacked adherence-related notes. Studies suggest oncologists may avoid documenting adverse effects or emotional distress due to time constraints or EHR design limitations [49,50]. This discrepancy underscores the need for improved EHR systems that support narrative input and patient-centred documentation. Findings from anonymous social platforms (Twitter/X, Reddit, forums) capture candid emotions that may not appear in identifiable EHR narratives. Accordingly, actions to surface patient voice should be stream-specific: conversational screening and peer-support signposting for social contexts, and concise narrative prompts and SDM documentation aids for EHRs. We caution against assuming full transferability of online sentiment to clinical records without tailored workflows and governance.

### 4.4. Interpretation of Findings

Using the Critical Appraisal Toolkit (CAT) [31], studies were rated for methodological rigour. High quality studies provided clearer links between patient sentiment and adherence, while lower quality studies often lacked transparency in NLP pipelines or adherence definitions. This informed the weighting of evidence in the synthesis. The synthesis demonstrates that emotional constructs such as fear, sadness, and spiritual pain are frequently detected in patient-authored content but are rarely documented in EHRs. Suboptimal communication and information gaps, as well as unclear concordance, are also recurrent issues. These findings suggest that both emotional and informational needs are central to understanding and addressing nonadherence.

While the included studies provide valuable insights, most focus on technical aspects of NLP or sentiment analysis rather than clinical application. As a result, direct evidence for specific nursing actions is limited. However, by integrating the themes and signals identified in the literature, we propose an interpretive framework to guide nursing assessment and intervention.

### 4.5. Implications for Practice

Findings suggest that oncologist communication, emotional support, and shared decision making are central to treatment concordance. NLP tools could be used to monitor patient sentiment and flag potential nonadherence risks. However, ethical considerations and interpretive caution are essential, especially when analyzing public online data [4,33]. Based on the synthesis of findings, we developed a practical framework mapping common emotion signals to likely patient behaviours and suggested concordance-oriented nursing responses. This framework is not a direct result from individual studies, but rather an expert synthesis intended to support nursing practice and future research.

This Table 3 should be interpreted as a practical synthesis, not as a direct empirical finding. It illustrates how emotional cues identified in patient narratives may signal specific adherence risks and suggests targeted nursing interventions. Such a framework may be particularly useful in settings where automated sentiment analysis is not yet available, supporting nurses in identifying and responding to patient needs in real time.

### 4.6. Limitations

The review is limited by the interpretive nature of discourse analysis and the paucity of NLP studies directly addressing nonadherence. Economic factors were excluded, and the focus was primarily on oral anticancer medication. Bias may arise from inferring nonadherence from related concepts, though this was mitigated by referencing prior work [4].

## 5. Conclusions

Despite limited direct evidence, this scoping review demonstrates that NLP studies of the cancer patient experience can infer treatment nonadherence and discordance through discourse analysis. Emotional needs may surpass informational needs, and oncologist attitudes and communication styles significantly influence concordance.

Most NLP studies focus on technical extraction rather than clinical application. However, sentiment analysis reveals unmet emotional needs and dissatisfaction that may impact adherence. EHR text mining shows that oncologist notes often lack detail on adverse effects, pain, or emotional distress, potentially due to time constraints or compliance-oriented design [49,50].

Improved oncologist communication and shared narratives in EHRs could enhance health literacy and treatment concordance. Future research should develop lexicons and NLP models that detect oncologist patient communication barriers and treatment discordance. A combined qualitative and NLP approach may offer deeper insight into the patient experience and support the aims of Patient-Centred Care [25,26].

## Figures and Tables

**Table 1:** Summary of cancer patient studies.

Author and Year	Method	Data Sources	Sample	Results	NLP Technique	Nonadherence/Inference
Clark et al., 2018 [13]	Quantitative	Twitter	∼5.3 million “breast cancer” related tweets. CAT: Strength = Moderate; Quality = Medium	Invisible patient-reported outcomes (iPROs) captured; positive experiences shared; fear of legislation causing loss of coverage.	Supervised machine learning combined with NLP	**Method of Inference:** Machine learning analysis of Twitter data. **Finding:** US context: fear of not receiving care salient. Analogous issues elsewhere may include lack of insurance and long waiting periods.
Turpen et al., 2019 [14]	Analysis of patient comments from surveys	Anonymous patient satisfaction surveys	25,161 surveys. CAT: Strength = Moderate; Quality = Medium	Statistically significant differences in language used for higher- vs. lower-rated physicians.	Frequency of 300 pre-selected n-grams	**Method of Inference:** Statistical analysis of language in patient comments. **Finding:** Time spent with patients matters. Longer physician times can enhance treatment adherence.
Lai et al., 2024 [15]	Quantitative (corpus-driven emotion analysis)	Cancer narratives corpus	—	Analyzing emotions in cancer narratives; corpus-driven approach highlights emotion categories relevant to adherence/concordance.	Corpus linguistics + NLP	**Method of Inference:** Emotion signals in narratives linked (inferred) to adherence risks. **Finding:** Fine-grained emotions can flag communication needs affecting adherence.
Mishra et al., 2013 [16]	Qualitative	Online conversations (prostate cancer patients)	34 websites; 3499 online conversations. CAT: Strength = Weak; Quality = Low	Patients often believed specialist information was biassed.	NLP to identify content and sentiment; ML; internal data dictionary	**Method of Inference:** NLP and manual review of online patient conversations. **Finding:** Perceived physician bias undermines trust and concordance. May influence treatment nonadherence.
Yin et al., 2017 [17]	Quantitative	Breastcancer.org	Retrospective study of 130,000 posts from 10,000 patients over 9 years. CAT: Strength = Moderate; Quality = Medium	Results correlated with traditional adherence surveys; emotions/personality readily detected online; scale increases relevance.	Logistic Regression (ML); emotion analysis	**Method of Inference:** Machine learning and emotion analysis of forum posts. **Finding:** Treatment completion associated with joy (and some disgust/sadness). Fear—especially of side effects—persistent and sometimes overlooked.
Li J et al., 2023 [21]	Qualitative	Weibo (Chinese social media platform)	150 written materials; 17 interviews; 6689 posts/comments. CAT: Strength = Weak; Quality = Low	Emotional lexicon with fine-grained categories; new perspective for recognizing emotions/needs; enables tailored emotional management plans.	Emotional lexicon; manual annotation (two general lexicons + BC-specific)	**Method of Inference:** Manual annotation of emotional expressions in patient narratives. **Finding:** Emotional expressions may predict adherence. Effective emotional management may be as important as information. Without support, intentional nonadherence is influenced by poor provider relationships.
Chichua et al., 2023 [22]	Quantitative	Public posts re: clinical trials; Reddit communities	129 cancer patients; 112 caregivers. CAT: Strength = Moderate; Quality = Medium	Fear identified as the highest emotion.	Keywords; NRC Emotion Lexicon; sentiment analysis	**Method of Inference:** Sentiment analysis of online clinical trial discussions. **Finding:** Sharing emotional experiences may increase fear of treatment withdrawal. Trust in the physician is an important factor (potential barrier) in clinical trials.
Freedman et al., 2016 [23]	Qualitative	Message boards; blogs; topical sites; content-sharing sites; social networks	1,024,041 social media posts about breast cancer treatment. CAT: Strength = Moderate; Quality = Medium	Fear was the most common emotional sentiment expressed.	Machine Learning (ConsumerSphere software)	**Method of Inference:** Machine learning analysis of social media posts. **Finding:** Fear of side effects dominant. Links to poor physician relationships and lack of treatment concordance. Rudeness a factor in poor communication.
Verberne et al., 2019 [24]	Posts labelled for empowerment and psychological processes	Forum for cancer patients and relatives	5534 messages in 1708 threads by 2071 users. CAT: Strength = Weak; Quality = Medium	The need for informational support exceeded emotional support.	LIWC	**Method of Inference:** Text mining of forum posts for empowerment and psychological processes. **Finding:** Lack of information may result in unintentional treatment nonadherence.
Cho et al., 2023 [34]	Quantitative (sentiment analysis and machine learning)	SVS member directory cross-referenced with a patient–physician review website	1799 vascular surgeons.CAT: Strength = Weak; Quality = Medium	The positivity/negativity of reviews largely related to words associated with the patient–doctor experience and pain.	Word-frequency assessments; multivariable analyses	**Method of Inference:** Analysis of patient–doctor communication in online reviews. **Finding:** Physician communication is a key factor influencing patient dissatisfaction and potentially nonadherence.
Masiero et al., 2024 [35]	Qualitative	Division of Senology, European Institute of Oncology	19 female metastatic breast cancer patients. CAT: Strength = Moderate; Quality = Medium	Themes: individual clinical pathway; barriers to adherence; resources to adherence; perception of new technologies.	Word-cloud plots; network analysis; sentiment analysis	**Method of Inference:** Thematic analysis of patient interviews and sentiment analysis. **Finding:** Patients experience fear related to clinical values. Ineffective communication and discontinuity of patient care are barriers to adherence.
Meksawasdichai et al., 2023 [36]	Quantitative	Retrospective tweets relevant to thyroid cancer	13,135 tweets. CAT: Strength = Moderate; Quality = Low	Twitter may provide an opportunity to improve patient–physician engagement or serve as a research data source.	Twitter scraping; sentiment analysis	**Method of Inference:** Sentiment analysis of patient tweets. **Finding:** Support in self-management is a main topic. Physicians may need to recommend online resources. Lack of support may lead to unintentional nonadherence.
Podina et al., 2023 [37]	Mixed methods	Reddit	187 users; 72,524 posts. CAT: Strength = Moderate; Quality = Medium	Short-term survivors are more likely to suffer depression than long-term; support in daily needs is lacking.	Lexicon and machine learning	**Method of Inference:** Mixed-methods analysis of Reddit posts. **Finding:** Need for online social media support. Patients do not typically discuss aspects of their disease with health professionals. Lack of trust and concordance influence treatment nonadherence.
Mazza et al., 2022 [38]	Non-interventional retrospective analysis of public social media	Social media posts (Twitter; patient forums)	76,456 conversations during 2018–2020. CAT: Strength = Moderate; Quality = Medium	Twitter was the most common platform; 61% authored by patients, 15% by friends/family, 14% by caregivers.	Predefined search string; content analysis	**Method of Inference:** Content analysis of social media posts. **Finding:** Poor communication and suboptimal relationships may influence treatment nonadherence.
Watanabe et al., 2022 [39]	Quantitative	Blog posts by patients with breast cancer in Japan	2272 blog posts. CAT: Strength = Moderate; Quality = Medium	Results helpful to identify worries and give timely social support.	BERT (context-aware NLP)	**Method of Inference:** NLP analysis of patient blogs. **Finding:** Concerns of patients change over time. Physician factors may influence treatment nonadherence.
Cercato et al., 2021 [40]	Qualitative (focus groups; thematic qualitative analysis)	National Cancer Institute (Rome)	31 cancer patients. CAT: Strength = Weak; Quality = Medium	Digital narrative medicine could improve oncologist relationship with greater patient input.	Narrative prompts for patients	**Method of Inference:** Thematic analysis of patient narratives. **Finding:** Lack of patient-centred care. Therapeutic alliance will promote adherence and improve treatment concordance. Neglect could result in unintentional nonadherence.
Vehviläinen-Julkunen et al., 2021 [41]	Mixed methods qualitative analysis (survey sentiment + focus groups)	National Cancer Patient Survey data; focus groups	92 participants (>65 years avg) and 7 focus groups (31 patients). CAT: Strength = Moderate; Quality = Medium	NLP automated sentiment analysis supported with focus groups informed the initial thematic analysis.	Automated sentiment analysis algorithm + focus groups	**Method of Inference:** Sentiment analysis of survey data and focus group input. **Finding:** Communication is vital to quality of care and in enhancing treatment adherence. Not actively soliciting patient feedback could result in nonadherence.
Law et al., 2021 [42]	Observational (voice/text analysis)	Online breast cancer forums	∼15,000 posts; 3906 unique users. CAT: Strength = Moderate; Quality = Medium	Engagement scores ranked relationships with HCPs as high; information needs are extremely high.	Lexicon-based analysis	**Method of Inference:** Lexicon-based analysis of forum posts. **Finding:** Lack of information may result in unintentional treatment nonadherence. Emotional needs may be of greater concern than informational needs.
Yerrapragada et al., 2021 [43]	Machine learning (insurance claims; population-based)	Commercial claims and encounters; Medicare claims	3022 breast cancer patients. CAT: Strength = Moderate; Quality = High	48% of patients were tamoxifen nonadherent.	Algorithms trained to predict nonadherence from claims features	**Method of Inference:** Machine learning prediction of nonadherence from claims data. **Finding:** High rates of nonadherence confirmed via ML. Results correlate with traditional surveys.
Moraliyage et al., 2021 [44]	Mixed methods (Quant ML/Qual NLP)	Online support groups; Twitter	2,469,822 tweets and 21,800 patient discussions. CAT: Strength = Moderate; Quality = Medium	Cancer patient information needs, expectations, mental health, and emotional wellbeing states can be extracted.	PRIME on Twitter; machine learning algorithms	**Method of Inference:** ML and NLP analysis of online support group and Twitter data. **Finding:** Fear underestimated in traditional surveys. May give rise to unintentional treatment nonadherence.
Balakrishnan, 2021 [45]	Quantitative	Breastcancer.org (3 OHS forums)	150,000 posts. CAT: Strength = Moderate; Quality = Medium	Deep learning more effective than machine learning.	Co-training: word embedding and sentiment embedding; domain-dependent lexicon	**Method of Inference:** Deep learning analysis of forum posts. **Finding:** Patients need current information. Emotions highest during the treatment phase. Information needs change along the disease trajectory.
Shah et al., 2021 [8]	Quantitative	National Health Service subsidiary website	53,724 online reviews of 3372 doctors. CAT: Strength = Moderate; Quality = Medium	Unstructured text mining identified key topics of satisfaction and dissatisfaction.	Sentic-LDA topic model; text mining	**Method of Inference:** Topic modelling of online reviews. **Finding:** Functional quality (communication; waiting times) may outweigh empathy. Attitude and communication dominate dissatisfaction.
Arditi et al., 2020 [46]	Cross-sectional survey (free-text comments)	Swiss Cancer Patients survey	844 patient comments. CAT: Strength = Moderate; Quality = Medium	Free text allows patients greater expression “in their own words”; enhances patient-centred care.	Computer-assisted textual analysis; manual expert corpus formatting	**Method of Inference:** Textual analysis of free-text survey comments. **Finding:** Poor communication and lack of information most common. Loneliness emerges as hidden factor—unmet support need.
Adikari et al., 2020 [47]	Quantitative/Qualitative	Conversations in ten international OCSGs	18,496 patients; 277,805 conversations. CAT: Strength = Moderate; Quality = Medium	Patients joining pre-treatment had improved emotions; long-term participation increased wellbeing; lower negative emotions after 12 months vs. post-treatment.	Validated AI techniques and NLP framework	**Method of Inference:** AI-based analysis of online support group conversations. **Finding:** Most patients sought treatment information initially. Transitioned to emotional support over time.
Mikal et al., 2019 [48]	Qualitative	Facebook	30 breast cancer survivors; ∼100,000 lines of data.CAT: Strength = Weak; Quality = Medium	Coding schema identified social support exchange at diagnosis and transition off therapy; social media support buffers stress.	Systematic coding (retrospective)	**Method of Inference:** Qualitative coding of Facebook support group data. **Finding:** Emotional support prioritized over informational support. Greater focus on emotional support may enhance adherence.

Abbreviations: NLP—Natural Language Processing; CAT—critical appraisal toolkit.

**Table 2:** Key patient side themes and prevalence.

Theme	% of Selected Sources
Unmet emotional needs (distress, fear, sadness, disgust, surprise) [15,17,22,23]	41%
Suboptimal information and communication (conflicting/insufficient info; perceived bias) [18,23,24]	50%
Concordance within PCC unclear (goal alignment, shared decision making indicators limited) [25,26,28]	55%
Online misinformation dynamics/perceived clinician bias [23,24,36]	(subset; qualitatively frequent)

Note: Percentages denote the fraction of included studies (numerator) in which each theme was identified during narrative synthesis over the total N=25 (denominator). They indicate signal consistency, not effect size. See Table 1 for study characteristics.

**Table 3:** Emotion signals, likely behaviours, and concordance-oriented responses. This interpretive framework is based on the synthesis of the literature and is intended as a practical guide for clinicians and researchers.

Emotion Signal	Likely Behaviour	Concordance Oriented Response
Fear/anxiety	Delay, dose skipping	SDM Team talk: acknowledge fear; distress screen; Option talk: clarify risks; Decision talk: values clarification.
Sadness/hopelessness	Withdrawal from self care	Psycho-oncology referral; follow up within 72 h; problem solving supports.
Disgust (AEs)	Refusal/cessation	AE management options; normalize toxicity; reframe goals.
Confusion/surprise	Inconsistent adherence	Teach back; simplified plan; bilingual materials.
Positive emotions	Sustained engagement	Reinforce self efficacy; document concordance; milestone planning.

## Data Availability

No new data were created or analyzed in this study. Data sharing is not applicable to this article.

## Public Involvement Statement

There was no public involvement in any aspect of this research.

## Guidelines and Standards Statement

This manuscript was drafted against the PRISMA-ScR checklist for reporting of scoping reviews.
